# A Novel Depression Recognition Network Based on Brain Functional Connectivity from Prefrontal Emotional State EEG

**DOI:** 10.3390/brainsci16070746

**Published:** 2026-07-14

**Authors:** Yubing Sun, Zijian Zhou, Jiaqi Sun, Wenjie Cui, Xinlei Hu, Yudan Lv, Guangda Liu

**Affiliations:** 1 Research Field of Medical Instruments and Bioinformation Processing, College of Instrumentation & Electrical Engineering, Jilin University, No. 938 West Democracy Street, Changchun 130061, China; sunyb22@mails.jlu.edu.cn (Y.S.); zjzhou21@mails.jlu.edu.cn (Z.Z.); sunjq19@mails.jlu.edu.cn (J.S.); cuiwj19@mails.jlu.edu.cn (W.C.); huxl19@mails.jlu.edu.cn (X.H.); 2 First Bethune Hospital of Jilin University, Changchun 130021, China; ydlv@jlu.edu.cn

**Keywords:** depression recognition, EEG, brain network topology, graph convolution network, dynamic temporal and spatial features

## Abstract

**Highlights:**

**What are the main findings?**

- The proposed DR-Net models dynamic spatiotemporal interactions in EEG-based depression recognition.
- Frontal EEG input reduction and GAN-driven augmentation improve data balance and model generalization.

**What are the implications of the main findings?**

- The model captures both local short-term interactions and global long-range dynamics, enabling depression-related biomarkers.
- The model establishes a comprehensive pipeline for multi-scale dynamic functional connectivity analysis of EEG.

**Abstract:**

**Objective:** Electroencephalogram (EEG) signals provide a noninvasive and objective method for detecting neural dysfunctions associated with depression. To address the limitations of limited sample sizes and inadequate modeling of spatiotemporal features, the study aims to develop an EEG-based depression recognition framework to capture dynamic patterns of neural activity related to depression. **Methods:** We propose DR-Net, a novel depression recognition framework integrating generative adversarial network (GAN), transition propagation graph convolution network (TPGCN), and transformer architectures. First, GAN-based data augmentation generates synthetic EEG samples that preserve the statistical properties of real data, improving data diversity and model generalization. Second, brain functional connectivity networks are constructed using the phase lag index, and the TPGCN models the dynamic propagation of information across channels, capturing the spatiotemporal evolution of brain network topologies beyond conventional static graph models. Concurrently, the transformer module employs self-attention to strengthen the modeling of long-range temporal dependencies. The framework also emphasizes the frontal lobe brain region, reducing input dimensionality while improving feature discriminability. **Results:** The proposed method achieved an accuracy of 98.7%, surpassing baseline and state-of-the-art methods, with no significant class bias. Validation on clinical datasets yielded an accuracy of 96.13%, demonstrating strong robustness and generalizability. **Conclusions:** By effectively modeling the spatiotemporal dynamics of EEG signals, DR-Net provides a reliable and generalizable approach to the objective diagnosis of depression and advances computational neurodiagnostic by integrating dynamic graph propagation, self-attention mechanisms, and data-efficient augmentation. These findings suggest that DR-Net may support future EEG-based clinical assessment of depression.

## 1. Introduction

Major depressive disorder (MDD) is a mental disorder characterized by high prevalence and chronicity [1]. It impairs individuals’ quality of life, social functioning and work productivity, and in severe cases, posing a serious risk to life [2]. Due to economic market downturns and escalating life pressures over the years, the number of individuals affected by depression has steadily increased. According to the data from the World Health Organization, approximately 4.0% of the global population are affected by depression. Depression remains a major global public health concern, and also represents an important contributor to non-fatal health loss and disability burden worldwide [3]. Depression imposes a substantial burden on both the healthcare system and the broader economy, necessitating urgent attention and prioritization [4].

Currently, the mainstream approach to diagnosing depression relies on clinical interviews conducted by healthcare professionals, supplemented by standardized assessment scales [5] such as the Mini-Mental State Examination (MMSE) [6], the Hamilton Depression Scale (HAMD) [7], the Diagnostic and Statistical Manual of Mental Disorders (DSM-5) [8], and the Beck Depression Inventory (BDI). Among them, MMSE is mainly used for cognitive screening. However, this method largely depends on the subjective judgment of clinicians and the active cooperation of patients, both of which introduce inherent limitations. Diagnostic bias is common, and ensuring objective consistency and accuracy in diagnostic outcomes remains challenging. Therefore, there is a pressing need to develop an objective and practical approach for the diagnosis of depression.

In recent years, objective methods for depression diagnosis have gradually attracted research attention [9], including neuroimaging techniques and physiological signal-based detection techniques. Common neuroimaging modalities include positron emission tomography (PET) and functional magnetic resonance imaging (fMRI). Although these techniques can provide valuable information on brain function and metabolism, their clinical application is limited by high cost, relatively low accessibility, and, in some cases, limited temporal resolution. Physiological signal detection techniques include the electrocardiogram (ECG), photoplethysmogram (PPG), and electroencephalogram (EEG), among others. Physiological signals serve as reliable indicators that effectively reflect the physical and psychological states of individuals. Biomarkers derived from physiological signals have increasingly attracted research interest. ECG can reflect the physiological responses associated with depression [10], but it provides limited information regarding the underlying neural mechanism of the disorder. PPG signals primarily reflect physiological activity in the vascular system [11] and demonstrate limited sensitivity for depression recognition. EEG is a non-invasive method with high temporal resolution, enabling the detection of subtle changes in brain electrical activity related to depression. These EEG alterations exhibit distinct neurophysiological patterns [12], providing potential physiological markers for understanding the neurobiological mechanism and supporting its early identification [13,14]. Due to its advantages of noninvasiveness, real-time monitoring capability, and low cost, EEG has increasingly become an important tool for EEG-based depression assessment and recognition.

EEG can reflect the neuro-electrophysiological activity of the brain and provide objective indicators for evaluating brain function. Studies have demonstrated that individuals with depression exhibit characteristic alterations in EEG signals, which establish a theoretical foundation for the application of EEG in the auxiliary diagnosis of depression. From the perspective of EEG power spectrum characteristics, brain electrical activity in individuals with depression exhibits significant band-specific patterns. Specifically, increased low-frequency band activity is observed in the frontal and temporal regions, while high-frequency band activity is reduced, with particularly notable differences in the β and α frequency bands [15]. For example, Mustufa et al. [16] used the Chaotic Owl Invasive Weed Search Optimization algorithm to select and optimize EEG power spectrum features in depressed patients, and the resulting classification model achieved an accuracy of 96%. Varun et al. [17] further validated abnormal EEG fluctuation patterns in depressed patients by extracting band power and correlation dimension as characteristic parameters, providing a reliable feature basis for disease monitoring. Beyond power spectrum characteristics, phase synchronization of EEG and interregional functional connectivity in MDD also exhibit abnormalities. Dell’Acqua et al. [18] found that the functional connectivity strength in the α and θ bands of EEG from individuals with severe depression was significantly higher than that in the healthy controls (HCs). Hasanzadeh et al. [19] calculated multiple EEG features including Lempel–Ziv complexity (LZC), power spectrum density, and fractal dimension, achieving a classification accuracy of 91.3%. Similarly, Yang et al. [20] employed LZC features derived from eyes-open EEG recordings for depression classification, with an accuracy of up to 86.58%.

Nevertheless, EEG signals exhibit pronounced nonlinearity, non-stationarity, and substantial inter-individual variability, making it difficult to fully characterize the physiological and pathological mechanisms of depression using conventional signal-level features. Accordingly, increasing research attention has shifted toward brain network topology, with EEG-based functional brain networks being constructed to characterize abnormal interregional interactions in patients with depression and to identify potential network-based biomarkers. Existing studies have shown that brain network characteristics can effectively reflect the abnormal patterns of neural activity associated with the depressed state. For instance, Shao et al. [21] quantitatively assessed the severity of depression by constructing a functional connectivity matrix, achieving robust discriminative performance. Zhang et al. [22] calculated the phase lag index (PLI) from 64-channel resting-state EEG to construct a functional connectivity matrix, and applied binary thresholding to the brain network to extract graph-theoretical metrics, thereby verifying the feasibility and effectiveness of using network-based features for depression classification.

Traditional machine learning algorithms used for EEG-based depression classification often rely heavily on the quality of manually engineered features, which may restrict the generalization performance. In contrast, deep learning methods emphasize the integrated processing of input and output, enabling automatic mapping between input signals and output labels, and can adaptively analyze relevant features of depressive brain activity. This demonstrates significant advantages in the field of depression classification. Graph convolutional neural networks (GCNs) represent an effective method for processing graph-structured data [23], capable of capturing spatial dependencies among nodes through parameter sharing and sparse connection mechanisms, thereby reducing model complexity and computational costs. Lan et al. [24] developed a GCN incorporating an attention mechanism to detect depression through topological information among EEG channels, with a sensitivity of 81.93%. Chen et al. [25] proposed a multi-scale dynamic GCN, achieving a depression classification accuracy of 90.51% through integrated analysis of EEG under positive and negative stimuli, thereby validating the effectiveness of GCNs in depression detection. Meanwhile, the transformer architecture demonstrates remarkable capabilities in modeling long-range dependencies and global information interaction, and has increasingly been applied to EEG-based emotion recognition and brain disorder detection tasks in recent years. Tigga et al. [26] proposed a transformer-based depression detection model using 60-channel EEG features, achieving a classification accuracy of 98.67%, demonstrating the potential of the transformer architecture in capturing global EEG features.

However, existing studies still face several critical scientific challenges. Predominantly, prevailing methodologies rely on static graph construction paradigms, which are insufficient to capture the dynamic reconfiguration of brain functional connectivity across task conditions or sliding temporal windows in MDD. From the perspective of temporal modeling, conventional Transformer architectures exhibit limited efficiency in processing long-sequence multichannel EEG signals. Moreover, their capacity to jointly encode spatial proximity and inter-channel dependencies remains constrained, which may hinder comprehensive modeling of deeply coupled spatiotemporal EEG features. Furthermore, EEG-based depression detection is generally affected by limited sample sizes, increasing the risk of overfitting and weakening model generalizability.

To address these limitations, we propose DR-Net, a novel deep learning framework for EEG-based depression classification, to enable collaborative and dynamic fusion of spatiotemporal representations. Specifically, to mitigate the impact of small sample size, a Generative Adversarial Network (GAN) is employed for data augmentation, generating samples that conform to the neurophysiological patterns of depression. We introduce a Transition Propagation Graph Convolution Network (TPGCN) module to model the time-varying topology of functional connectivity through adaptive neighborhood refinement. Furthermore, we design a spatiotemporal interactive Transformer module, in which spatial graph structure explicitly guides temporal feature selection, while temporal dynamics reciprocally modulate spatial edge weights. Collectively, these components overcome the spatiotemporal decoupling bottleneck in existing EEG decoding approaches, yielding improvements in classification performance, clinical interpretability, and robustness.

The contributions of the current study are as follows.

(1) A novel deep learning model, DR-Net, was proposed to implement EEG-based depression recognition under emotional stimulation tasks. The model effectively captured dynamic spatiotemporal interaction patterns in EEG signals, enabled the transition from independent channels analysis to multi-channel dynamic functional connectivity, and comprehensively explored the potential neural dynamic features in the electrophysiological activity associated with depression.

(2) A task-driven frontal-region-focused EEG modeling strategy is introduced to reduce input redundancy while preserving depression-relevant discriminative information, thereby improving training efficiency and feature representation capability. To address the challenges of limited EEG sample size and imbalanced class distribution, a GAN was introduced for data augmentation and integrated with DR-Net to establish a closed-loop optimization of “data augmentation—feature extraction”, effectively enhancing the generalization ability of the model.

(3) Our framework integrated the hierarchical spatiotemporal fusion architecture of TPGCN and Transformer, simultaneously capturing both local channel interactions within short time windows and global dynamic trends over long-term scales in depressive EEG signals. This enables the collaborative representation and deep fusion of multi-scale spatio-temporal dependencies, thereby providing a more comprehensive representation of multi-level biological markers related to depression.

The organizational structure of the remaining part of this paper is as follows. The second part briefly describes the source of the subjects and introduces the overall process and detailed content of identifying depression using EEG signals under the emotional stimulation task. Section 3 describes and analyzes the experimental results obtained from the proposed model, with comparative evaluation and validation against existing studies. Part IV summarizes and discusses the key findings of this study, along with implications and future research directions.

## 2. Materials and Methods

**Figure 1:**
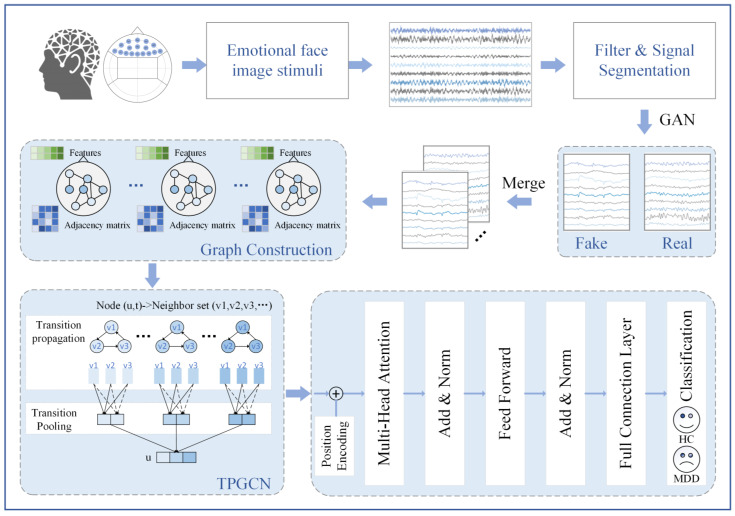
The overall framework of DR-Net for EEG-based depression recognition under emotional states.

To facilitate EEG analysis of depression under emotional stimulation tasks, the overall research framework proposed in this paper is shown in Figure 1. First, frontal EEG signals are collected from subjects during the emotional stimulation task and subsequently filtered to remove noise and artifacts, thereby improving signal quality. Then, to enrich the training samples and enhance model generalization, a GAN-based data augmentation strategy is subsequently applied to the training EEG data. Subsequently, features are derived from the preprocessed EEG signals, and inter-channel interaction relationships are computed based on the PLI to construct the functional brain network. Finally, the constructed graphs are fed into the DR-Net model proposed in this paper to extract spatiotemporal multiscale features from the EEG, thereby supporting the identification of potentially depression-related neurophysiological patterns and the classification of depressive states.

### 2.1. Subjects

#### 2.1.1. MODMA Dataset

Subjects were selected from the Multimodal Open Data Set for Mental Disorder Analysis (MODMA) [27], published by Lanzhou University. The dataset contains 128-electrode EEG recordings from 53 participants under both resting and stimulated conditions. All participants were clinically diagnosed, had no history of substance dependence or other neurological disorders, and were right-handed and carefully screened by board-certified psychiatrists. The emotional stimulation experiment included three categories of facial expressions: fear-neutral, sad-neutral and happy-neutral (abbreviated as fear, sad, and happy, respectively). EEG signals were recorded through a 128-channel HydroCel Geodesic Sensor Net in conjunction with Net Station acquisition software (version 4.5.4). Additional details regarding sampling rates and other parameters are summarized in Table 1.

#### 2.1.2. Clinical Data

The participants were recruited from a Grade A tertiary hospital, with a total of 52 individuals enrolled. The recruitment procedure was conducted through the Department of Psychiatry. Potential participants were initially screened by experienced psychiatrists according to the predefined inclusion and exclusion criteria. Eligible individuals were then informed of the study purpose, experimental procedures, and related requirements, and those who agreed to participate provided written informed consent before EEG data acquisition. The inclusion criteria required participants to be between 18 and 55 years of age and to have received at least primary school education. Participants were excluded if they had taken any psychotropic medication within two weeks before the experiment; received electroconvulsive therapy, transcranial stimulation, or other related treatments within the past three months; had a history of drug or alcohol abuse; had any neurological disorder; had severe head injury; or presented with suicidal tendency or acute physical illness. All participants underwent structured clinical interviews conducted by experienced psychiatrists, and their depression severity was evaluated using the HAMD to ensure the clinical validity of group classification.

The experimental design consisted of two parts. The first part was an emotional induction task, in which participants were presented with emotional facial images to elicit distinct emotional states. The emotional face images used encompassed four emotion categories: happy, sad, fear, and neutral. Images from different emotional categories were presented randomly and differentiated through specific marker signals. The second part involved EEG data collection. While emotional stimuli were being presented, EEG signals were acquired in real time using the NeuroScan EEG recording system (Compumedics, Charlotte, NC, USA), with a focus on monitoring neural activity in the frontal lobe to capture EEG responses related to the depressive state. The sampling rate and other information are detailed in Table 1.

### 2.2. Preliminary

#### 2.2.1. EEG Preprocessing

**Figure 2:**
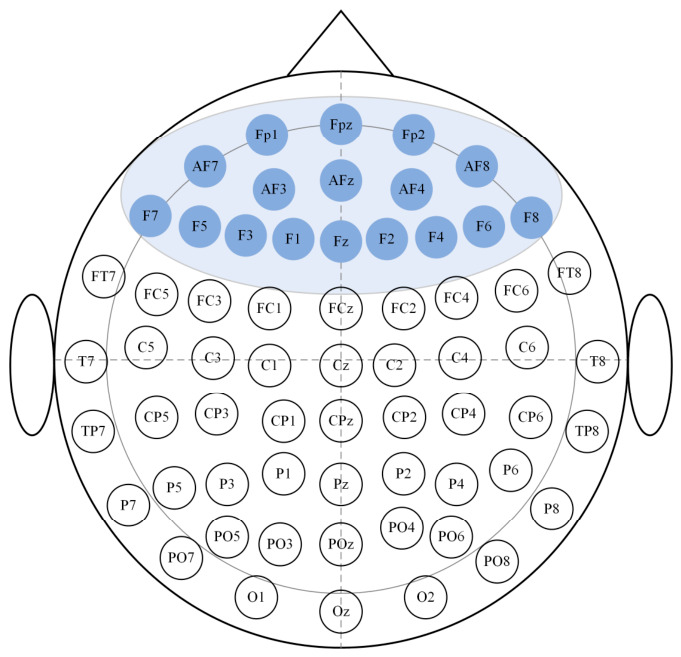
Regional division of the brain and distribution of prefrontal electrodes.

Given a set $Gh={\left\{g^{i}\right\}}_{i=1}^{Gh}$ comprising Gh subjects, each subject is represented from a graph-theoretic perspective as $g^{i}\;=\;(V^{i},E^{i},X^{i})$. Here, $V^{i}\;=\;\left\{v_{1}^{i},v_{2}^{i},\ldots v_{\mathrm{Ch}}^{i}\right\}$ denotes the node set, corresponding to the EEG electrodes in this study, $E^{i}$ represents the edge set, defined as the connection strength between nodes, and $X^{i}\;=\;\left\{x_{1}^{i},x_{2}^{i},\ldots ,x_{\mathrm{Ch}}^{i}\right\}$ indicates the node feature set, where Ch represents the number of EEG channels. The 17 EEG channels located in the prefrontal lobe of each participant were designed as nodes in this study, with their spatial arrangement detailed in Figure 2.

During the acquisition of brain electrical signals, artifacts such as eye movements, electromyography (EMG), and electrooculography activity may be introduced, leading to degraded signal quality and adversely affecting subsequent processes. Therefore, the original EEG signals are initially preprocessed. A band-pass filter with a frequency range of 0.5 to 40 Hz is applied to eliminate high-frequency noise and low-frequency drift. Then, independent component analysis is employed to decompose and reconstruct the EEG signals. A trained and experienced EEG expert was invited to identify components containing prominent physiological or movement-related artifacts. Components that were severely contaminated by artifacts were excluded before signal reconstruction, thereby effectively reducing the impact of ocular and EMG artifacts.

Given the limited sample size, a GAN is employed to perform data augmentation, thereby expanding the training dataset and enhancing the generalization capability of the model. The network comprises a generator G and a discriminator D [28]. The G takes a random noise vector z as input and generates synthetic samples G(z), while the D evaluates the samples by distinguishing between real and generated samples and computing the corresponding adversarial loss. The two parts are iteratively optimized through a competitive training process until the discriminator can no longer reliably differentiate between real and synthetic data, indicating convergence of the training procedure. The objective function of the GAN is defined as:

(1) $$\min_{G}\max_{D}V\left(D,G\right)\;={\;E}_{x\sim \mathrm{pdata}}\left(x\right)\left[\log\left(D\left(x\right)\right)\right]\;+{\;E}_{z\sim \mathrm{pz}\left(z\right)}\left[\log\left(1-D\left(G\left(z\right)\right)\right)\right]$$

where pz(z) denotes the distribution of the noise vector, and pdata(x) represents the distribution of the actual data. Through iterative optimization, signals that are statistically similar to the actual EEG signals are generated and subsequently used for model training and validation. In our study, the augmentation ratio was set to 1:1 for each class. GAN-based augmentation was performed independently within each cross-validation fold, and the generated samples were used only for model training within the corresponding fold.

#### 2.2.2. Feature Extraction

Brain electrical signals inherently exhibit nonlinear and non-stationary characteristics, and their raw waveforms do not directly reflect the complex dynamic patterns of neural activity associated with depression. To comprehensively characterize EEG features under the depressed state, we performed feature extraction and analysis of EEG from four domains: time domain, frequency domain, nonlinear dynamics, and graph-theoretical features. Specifically, time-domain features are employed to reflect the statistical distribution properties of the signals, including the mean, standard deviation, variance, Hjorth parameters, skewness, and kurtosis. Frequency-domain features can describe the signal energy distribution across various frequency bands, and relevant metrics are extracted using spectral analysis and power spectral density estimation techniques. Nonlinear features are used to capture the chaotic and complex dynamics of EEG signals, including detrended fluctuation analysis (DFA) and C0 complexity. DFA [29] is used to characterize long-range temporal correlations and scale-free temporal organization of EEG activity. C0 complexity [30] was selected to quantify nonlinear signal complexity and irregularity. The computation of graph-theoretical features is based on the constructed functional connectivity network, which characterizes the topological organization among channels, with key metrics including clustering coefficient (CC) and global efficiency (GE), which can characterize local segregation and global integration, respectively [31]. The calculation procedures for time-domain and frequency-domain features are presented in Table 2, while definitions and computational methods for other essential features are provided as follows.

Detrended fluctuation analysis (DFA): A mathematical method used to analyze stochastic processes by estimating the correlation properties of time series data.

(2) $$F\left(n\right)=\sqrt{\frac{1}{N}\sum_{k=1}^{N}\left(X\left(k\right)-Y_{n}\left(k\right)\right)}$$

where F(n) denotes the fluctuation function. $X\left(k\right)=\sum_{i=1}^{k}\left(x\left(i\right)-mean(x)\right)$. $Y_{n}\left(k\right)$ represents the least squares fit of X(k), obtained by dividing the time series into n equally sized segments and performing linear regression within each segment. By varying the window length and thus obtaining different values of F(n), the feature results can be characterized through the logarithmic relationship between F(n) and n.

(3) $$\mathrm{DFA}=\log_{n}\left(F\left(n\right)\right)$$

C0 complexity (C0): Defines the ratio of irregularity to regularity and is capable of quantifying the complexity and randomness inherent in time series data.

The average amplitude of the power spectrum of the time series x(n):

(4) $$M\;=\;\frac{1}{N}\sum_{k=0}^{N-1}{\left|X\left(k\right)\right|}^{2}$$

where X(k) denotes the Fourier transform of x(n). Construct the new spectrum:

(5) $$Y\left(k\right)\;=\;\left\{\begin{array}{c} X\left(k\right)\\ 0 \end{array}\;\begin{array}{c} {\left|X\left(k\right)\right|}^{2}\;>\;M\\ {\left|X\left(k\right)\right|}^{2}\;<\;M \end{array}\right.$$

The inverse Fourier transform is applied to Y(k) to obtain y(n), from which C0 complexity can be computed.

(6) $$C0=\frac{A_{1}}{A_{0}}=\frac{\sum_{n=0}^{N-1}{\left|x\left(n\right)-y\left(n\right)\right|}^{2}}{\sum_{n=0}^{N-1}{\left|x\left(n\right)\right|}^{2}}$$

where $A_{1}$ and $A_{0}$ denote the power values corresponding to the irregular and regular components of the time series x(n), respectively.

Clustering coefficient (CC): A measure of functional separation [32] that quantifies the local organization of a network by capturing the propensity of nodes to form interconnected clusters or local triangular structures.

(7) $$C_{i}=\frac{2t_{i}}{k_{i}{\left(k_{i}-1\right)}^{\prime }}$$

where $t_{i}=\frac{1}{2}\sum_{j,h\in N}{\left(w_{\mathrm{ij}}w_{\mathrm{ih}}w_{\mathrm{jh}}\right)}^{\frac{1}{3}}$ represents the geometric mean of the number of triangles centered at node i, and $k_{i}=\sum_{j\in N}w_{\mathrm{ij}}$ denotes the degree of that node. The CC of the entire network is defined as the arithmetic average of the clustering coefficients across all nodes.

(8) $$\mathrm{CC}=\frac{1}{N}\sum_{i\in N}C_{i}$$

Global efficiency (GE): Denotes the inverse of the mean shortest path length between all pairs of nodes, which is employed as an indicator for evaluating the efficiency of information propagation in complex networks. The CC and GE were computed through the Brain Connectivity Toolbox [33] to enable a systematic evaluation of the topological properties of the network. Specifically, this indicator assesses the efficiency of information exchange between nodes through mathematical formulations. The average shortest path length represents the mean distance between any two nodes, and its reciprocal offers a more intuitive characterization of the network’s overall capacity for information dissemination. Higher values indicate enhanced connectivity, smoother information flow, and greater efficiency of interaction among nodes.

#### 2.2.3. Construction of Brain Functional Matrices

To comprehensively account for the influence of neural connectivity on depression identification, the PLI is computed to construct the prefrontal functional connectivity network in individuals with depression. The procedure for calculating the PLI is outlined as follows.

At time t, given a pair of EEG data $x_{j}\left(t\right)$ and $x_{k}\left(t\right)$, the phase difference is mathematically defined as

(9) $$\left|\Delta \varphi_{n,m}\left(t\right)\right|=\left|n\varphi_{j}\left(t\right)-m\varphi_{k}\left(t\right)\right|$$

where n and m are both equal to 1 in typical neuroscience applications. $\varphi_{j}\left(t\right)$ and $\varphi_{k}\left(t\right)$ represent the instantaneous phase components of the signals $x_{j}\left(t\right)$ and $x_{k}\left(t\right)$, respectively, which are derived through the following computational equation:

(10) $$\varphi_{j}\left(t\right)\;=\;\arctan\frac{{\tilde{x}}_{j}\left(t\right)}{x_{j}\left(t\right)}$$

where ${\tilde{x}}_{j}\left(t\right)$ is the Hilbert transform of $x_{j}\left(t\right)$.

(11) $${\tilde{x}}_{j}\left(t\right)=\frac{1}{\pi }\mathrm{PV}\int_{\text{-}\infty }^{\infty }\frac{x_{j}\left(\xi \right)}{t-\xi }d\xi$$

where PV denotes the Cauchy principal value. The PLI between $x_{j}\left(t\right)$ and $x_{k}\left(t\right)$ can be defined as:

(12) $${\mathrm{PLI}}_{\mathrm{jk}}\;=\;\left|\frac{1}{L}\sum_{i=0}^{L-1}\mathrm{sign}\left(\Delta \varphi \left(t_{i}\right)\right)\right|,\;0\;\leq \;{\mathrm{PLI}}_{\mathrm{jk}\;}\leq \;1$$

where L represents the number of data samples, and sign denotes the signal function.

### 2.3. DR-Net for Depression Recognition

#### 2.3.1. Transition Propagation Graph Convolution Network

During the EEG testing for depression in the task state, individuals with depression are influenced by experimental stimuli and procedures, which often leads to signal fluctuations. Therefore, it is necessary to model the dynamic spatial dependency among EEG channels rather than assuming a fixed and static connectivity pattern. To illustrate this approach, a novel transition propagation graph convolutional network (TPGCN) is developed based on the transition propagation graph neural network proposed by Zheng et al. [34], which enhances the traditional graph convolutional neural network to explicitly model the evolving propagation of information between EEG channels. It captures the dynamic spatial relationships between brain channels by accounting for the mutual influence among diverse features across multiple channels, thoroughly exploring node-wise correlations, fully leveraging the rich spatiotemporal dependencies among multiple channels, and enhancing the model’s interpretability regarding interactions between brain electrodes.

TPGCN comprises three modules: sequence translation, transition propagation, and bilevel graph convolution. These modules are designed for the construction of directed transition graphs, the encoding of graph transition structures, and the extraction of salient information from transition graphs, respectively. For any node u and timestamp t, the historical interaction representation is denoted as $S_{u,t}\;=\;\{s_{1},s_{2},\ldots ,s_{m}\}$, where $s_{i}\;=\;(u,v_{i},t_{i})$ represents the interaction between node u and its neighbor node $v_{i}$ at time $t_{i}$. The cosine temporal kernel function is utilized to encode the temporal differences, effectively capturing meaningful patterns in the time dimension, and is defined as:

(13) $$\phi \left(\Delta t\right)\;=\;\mathrm{concat}\left(\cos\left(\omega_{1}\Delta t\right),\ldots ,\cos\left(\omega_{x_{t}}\Delta t\right)\right)$$

where $\Delta t\;=\;t-t_{l}$ denotes the time interval between the predicted timestamp and the edge timestamp. $\omega_{1}$ represents the frequency adaptive parameter of the trainable cosine function, and $x_{t}$ represents the dimension of the output carrier. By integrating the aforementioned node features, interaction features and temporal features, we can obtain:

(14) $$Z_{N_{u,t}}^{0}\;=\;W\cdot \mathrm{ReLU}\left(W_{n}H_{N_{u,t}}^{l}\;+{\;W}_{e}B_{u,t}H_{s}\right)+b$$

where $H_{N_{u,t}}^{l}$ represents the features of the neighboring nodes at the l-th layer, $B_{u,t}$ is the feature correlation matrix, and $H_{s}={\left[{\tilde{e}}_{1},{\tilde{e}}_{2},\ldots ,{\tilde{e}}_{m}\right]}^{T}$ represents the interaction feature matrix, with ${\tilde{e}}_{i}\;=\;\mathrm{concat}(e_{i},\phi \left(\Delta t_{i}\right))$. $e_{i}$ is the original interaction feature, and W, $W_{n}$, $W_{e}$, and b are all trainable parameters.

The graph propagation module aims to encode dynamic characteristics within the transition graph. It utilizes a convolutional neural network (CNN) to enhance the nonlinear feature representation, and subsequently integrates the transition matrix to propagate information:

(15) $${\tilde{H}}_{N}^{k+1}\;=\;{\tilde{A}}_{u,t}\mathrm{CNN}\left(H_{N}^{k}\right)$$

where k represents the number of propagation steps, and $H_{N}^{k}$ denotes the embedding of the neighboring node at the k-th step.

Finally, a two-layer graph convolution and pooling architecture is utilized to extract salient features, thereby producing the aggregated interaction representation of brain nodes, and being fed into the subsequent Transformer module for temporal dependency modeling.

#### 2.3.2. Transformer Encoder Network

Although TPGCN effectively captures the dynamic spatial dependencies among EEG channels, depression-related EEG patterns also exhibit strong temporal dynamics, such as long-range correlations and non-stationary fluctuations across time. Therefore, it is essential to further capture the temporal dependency of the extracted spatial representations. The transformer network is a model architecture based on an encoder–decoder framework [35] that employs the transformer mechanism to capture long-range dependencies in EEG sequences. It models the relationships between distant time steps in EEG by using the self-attention mechanism, thereby overcoming the limitations of LSTM in capturing long-range temporal dependencies and enabling more effective integration of temporal information from EEG signals. To capture the temporal dependencies of depression-related EEG features, we employ positional encoding and encoder layers within the transformer to model sequential information and learn temporal embeddings of features at each time step. This enables the incorporation of temporal dynamics into the sequence representation and improve the accuracy of the model in disease identification.

First, for the output $H_{t}$ at each time step t, the outputs are stacked into a matrix $H\in R^{T\times D^{\prime }}$, where $D^{\prime }$ is the output feature dimension of the graph convolution layer. A position encoding matrix $\mathrm{PE}\in R^{T\times D^{\prime }}$ is then initialized and added to H via element-wise summation, yielding an enhanced representation $H^{\prime }$, which serves as the input to the encoder for further learning.

(16) $$H^{\prime }=H+\mathrm{PE}$$

The encoder achieves efficient scaling for long sequences by processing distant dependencies in parallel by leveraging multi-head self-attention mechanisms and feed-forward neural networks. The core computational process is as follows.

The time series H′, obtained by positional encoding, is used as input and projected into three distinct subspaces: the query subspace $Q\;=\;H^{\prime }W_{Q}$, the key subspace $K\;=\;H^{\prime }W_{K}$, and the value subspace $V\;=\;H^{\prime }W_{V}$. The encoder computes the self-attention scores between the query and key vectors [36], and the softmax function is subsequently applied to derive the normalized attention weights.

(17) $$M=\mathrm{Attention}\left(Q,K,V\right)=\mathrm{softmax}\left(\frac{QK^{T}}{\sqrt{d_{k}}}\right)V$$

where $W_{Q}$, $W_{K}$, and $W_{V}$ denote the learnable linear transformation matrices for the query, key and value, respectively, which are shared across all nodes, and $d_{k}$ represents the dimensionality of the key [37].

The multi-head attention mechanism explores patterns across different representation subspaces by employing distinct sets of learnable weight matrices. The computation is formulated as follows:

$$\mathrm{MultiHead}\left(Q,\;K,\;V\right)\;=\;\mathrm{Concat}({\mathrm{head}}_{1},\ldots ,{\mathrm{head}}_{h})W^{O}$$

(18) $$\mathrm{where}\;{\mathrm{head}}_{i}=\mathrm{Attention}\left(QW_{i}^{Q},KW_{i}^{K},VW_{i}^{V}\right)$$

where $W_{i}^{Q}\in R^{d_{\mathrm{model}}\times d_{k}},W_{i}^{K}\in R^{d_{\mathrm{model}}\times d_{k}},W_{i}^{V}\in R^{d_{\mathrm{model}}\times d_{v}}$, and $W^{O}\in R^{hd_{v}\times d_{\mathrm{model}}}$.

Feedforward networks and normalization layers are employed to capture interactions among latent features. The outputs from different attention heads are processed in parallel to enhance training efficiency. This feedforward processing is implemented through two linear transformations followed by the ReLU activation function. The output of the encoder is subsequently fed into a fully connected layer, where the representation vector is normalized via the softmax function to generate class probabilities, which are then used for the final classification task.

#### 2.3.3. Loss Function

To ensure effective parameter updating of the DR-Net model during training, the cross-entropy loss function is employed to compute the prediction error. This approach emphasizes the accuracy of the predicted probability for the correct class label, which is crucial for accurately distinguishing between MDD and HCs in the present study.

(19) $$\mathrm{Loss}=-\left(\mathrm{ylog}\hat{y}+\left(1-y\right)\log\left(1-\hat{y}\right)\right)$$

Algorithm 1 describes the flow of our method as follows:
**Algorithm 1.** The description of DR-Net for EEG Depression RecognitionRequire: EEG data X, labels Y, learning rate *lr*, batch size B, and training epoch e. Ensure: The model prediction $\hat{Y}$. 1. Preprocess and segment EEG signals; 2. Augment EEG data through the GAN; 3. Calculate node features and adjacency matrix to generate the graph; 4. Construct the EEG dynamic graph and input it into the TPGCN to update the node features; 5. Convert the output of TPGCN to the data dimension; 6. Fuse the temporal dependencies of long-term time steps based on learned weights through the transformer module; 7. Input the long-term temporal features into the fully connected layer; 8. Perform gradient descent using the loss function; 9. Repeat steps 2–8 to train the model; 10. Return;

### 2.4. Implementation Details

To assess the effectiveness of the proposed method, five evaluation metrics were utilized to quantitatively evaluate the classification performance of DR-Net. EEG segments derived from the same subject were not assigned to multiple data partitions within the same fold. The model was trained for 50 epochs using the Adam optimizer with a learning rate of 0.001. A dropout rate of 0.5 was applied during training to reduce overfitting and improve model generalization.

(20) $$\mathrm{Accuracy}=\frac{\mathrm{TP}+\mathrm{TN}}{\mathrm{TP}+\mathrm{TN}+\mathrm{FP}+\mathrm{FN}}$$

(21) $$\mathrm{Precision}=\frac{\mathrm{TP}}{\mathrm{TP}+\mathrm{FP}}$$

(22) $$\mathrm{Sensitivity}=\frac{\mathrm{TP}}{\mathrm{TP}+\mathrm{FN}}$$

(23) $$\mathrm{Specificity}=\frac{\mathrm{TN}}{\mathrm{FP}+\mathrm{TN}}$$

(24) $$F1\text{-}\mathrm{score}=2\;\times \;\frac{\mathrm{Precision}\;\times \;\mathrm{Sensitivity}}{\mathrm{Precision}+\mathrm{Sensitivity}}=\frac{2*\mathrm{TP}}{2\mathrm{TP}+\mathrm{FP}+\mathrm{FN}}$$

The proposed DR-Net was developed in Python 3.8.5 based on the PyTorch framework. The experiments were conducted on a computer equipped with an NVIDIA GeForce RTX 4060 graphics card and a 12th Gen Intel^®^ Core™ i5-12600KF CPU.

## 3. Experimental Results and Discussion

### 3.1. Analysis of EEG Features About Depression and Health

**Figure 3:**
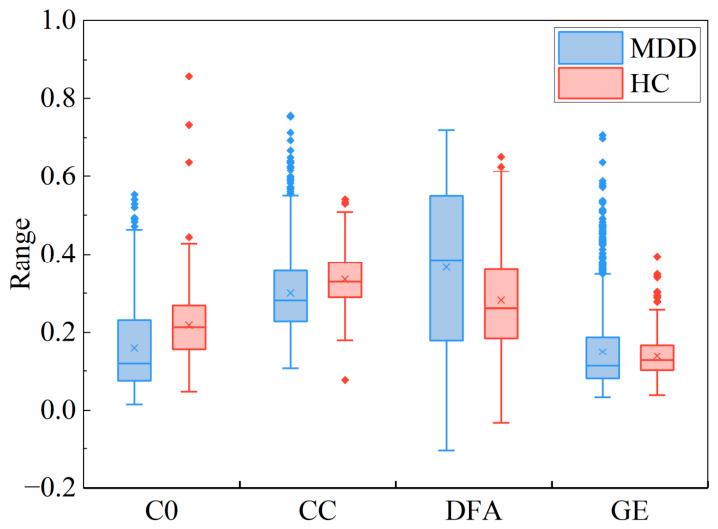
The boxplots of the detrended fluctuation analysis, C0 complexity, clustering coefficient, and global efficiency of MDD and HC subjects at frontal areas of the scalp.

The primary objective of this section was to thoroughly investigate the differences in EEG signal characteristics within the prefrontal cortex between individuals with depression and HCs. To achieve this, nonlinear dynamic properties and graph-theoretical features were analyzed. Figure 3 presents a visual summary of several key metrics derived from the EEG data, including DFA, C0 complexity, clustering coefficients, and global efficiency. The visualization revealed distinct descriptive differences between individuals with depression and healthy controls. Specifically, MDD individuals exhibited higher DFA values and increased clustering coefficients, indicating enhanced local connectivity and greater persistence in neural activity patterns. In contrast, the MDD group showed reduced C0 complexity and lower global efficiency, suggesting potentially diminished signal complexity and impaired global information integration across distributed brain regions. These findings collectively suggest that the nonlinear dynamic features and network-level properties examined in this study may be sensitive to neurophysiological alterations associated with depression. Such insights may contribute to a deeper understanding of the underlying neural mechanisms in depression and could assess their potential value as EEG-based biomarkers for depression assessment or treatment monitoring.

**Figure 4:**
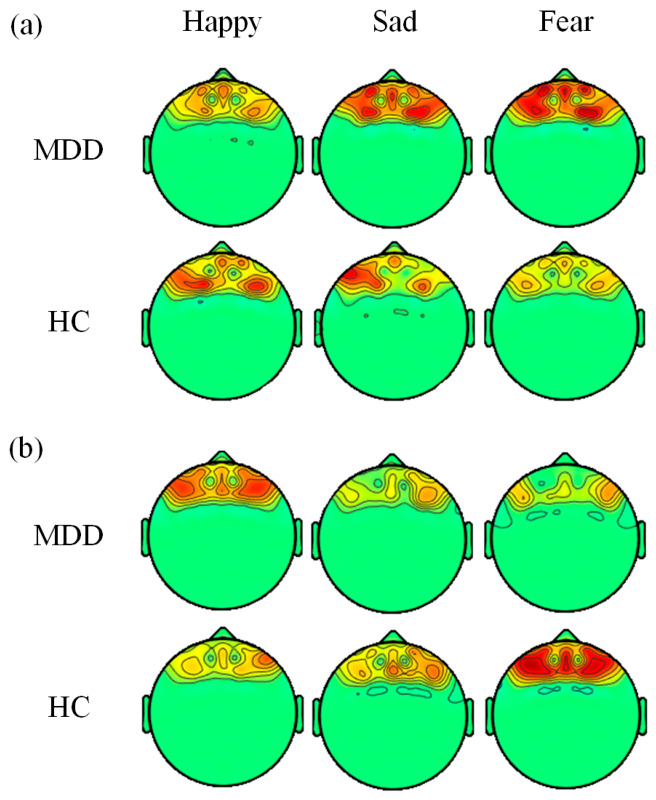
Prefrontal C0 complexity and DFA topography of subjects with different emotional stimuli. (**a**) C0 complexity. (**b**) DFA.

**Figure 5:**
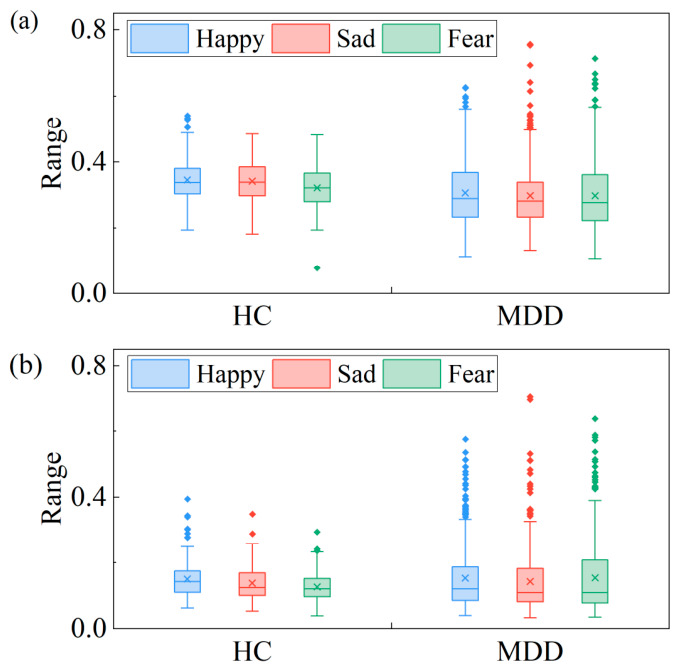
Comparative box plots of prefrontal clustering coefficients and global efficiency in subjects with different emotional stimuli. (**a**) Clustering coefficients. (**b**) Global efficiency.

Figure 4 presents topographic maps comparing prefrontal C0 complexity and DFA between MDD and HCs under different emotional stimulus conditions. These visualizations provide an intuitive spatial representation of brain activity patterns in response to various emotional cues. Figure 5 displays box plots of the prefrontal clustering coefficient and global efficiency in MDD and HCs across varying emotional stimuli, offering a descriptive comparison of network-level EEG features related to local connectivity and global information integration.

The results indicate that individuals with depression exhibit more pronounced fluctuations in neurophysiological features in response to negative emotional stimuli, such as fear and sadness. This heightened sensitivity is particularly evident in the prefrontal cortex, a brain region closely associated with emotional regulation and cognitive control. This suggests that negative emotional stimuli elicit stronger neural responses in depressed individuals, leading to greater variability in their electrophysiological signals. These variations in brain activity may reflect potential disruptions in emotional processing and self-regulation mechanisms. Furthermore, the observed differences in complexity and efficiency metrics between MDD and HCs provide useful information for understanding neurophysiological alterations. The altered patterns of brain dynamics in response to emotional stimuli suggest potential changes in functional brain organization associated with depression. These findings may provide preliminary evidence for EEG-based feature analysis in depression recognition and support further investigation of potential neurophysiological markers for depression assessment.

### 3.2. Analysis of Multiscale and Multichannel Topology About Depression and Health

**Figure 6:**
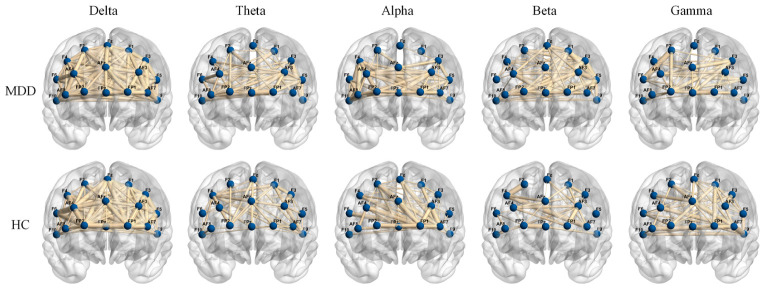
Visualization of multi-scale functional connectivity in MDD and HC subjects.

It is essential to investigate depression from a multi-channel and multi-scale perspective. The 10–20-electrode placement system enables the acquisition of bioelectric signals from multiple brain regions, thereby reflecting real-time neural activity. Given that the prefrontal cortex exhibits more pronounced responses in individuals with depression, this study aims to examine the interactions among prefrontal EEG signals, analyze the correlations between signals recorded from different electrodes, and quantify these functional connections using PLI. To further illustrate these patterns, prefrontal channel relationships were visualized. Figure 6 presents prefrontal multiband functional connectivity for the HCs and depression groups with happy, sad, and fearful emotional stimuli.

In each subplot, the functional connectivity strength between electrodes is represented by the thickness of the connecting lines. Thicker lines indicate stronger inter-channel phase synchronization, whereas thinner lines indicate weaker correlations. Descriptively, healthy controls exhibit stronger functional connectivity in the delta, theta, and full frequency band. The visualization of functional connectivity provides an intuitive representation of channel interactions, which may help characterize EEG connectivity patterns associated with MDD.

### 3.3. Results and Analysis of the Classifiers and Methods

**Figure 7:**
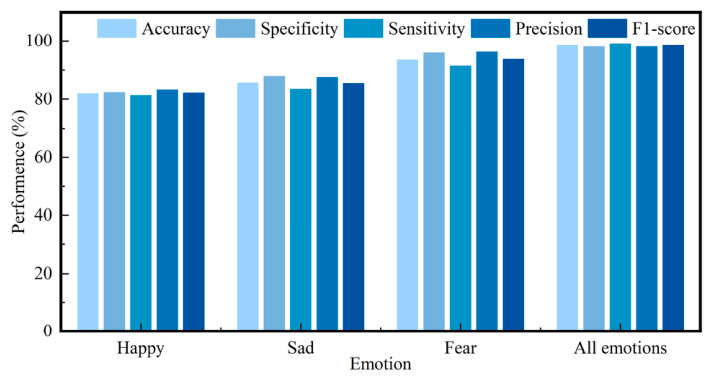
Depression identification results for different emotional stimulus tasks.

Existing studies have demonstrated that individuals with depression exhibit heightened sensitivity to negative emotional stimuli [38]. To validate this phenomenon, this study investigates depression from multiple emotional stimulus perspectives by employing distinct single emotional stimulus tasks, including happy, sad, and fear conditions. The experimental performance metrics are presented in Figure 7. The accuracy of depression recognition based on fearful stimuli reached 93.70%, which is significantly higher than the 82.04% accuracy obtained using happy stimuli. This indicates that the differentiation between individuals with depression and HCs is more pronounced under negative emotional stimuli. These findings suggest that negative emotional stimuli are more effective in eliciting neural fluctuations in individuals with depression, thereby enhancing brain responses and increasing the likelihood of capturing depression-related sensitive information. These results are consistent with previous research and offer further support and reference for future in-depth studies on depression identification.

The analysis of the experimental results revealed that the use of composite emotional stimuli yielded higher depression recognition accuracy compared to single emotional stimulus tasks. This finding suggests that individuals with depression may exhibit abnormalities in brain connectivity during dynamic emotional processing, and that the comparison across multiple emotional stimuli can enhance the detection of such aberrant features [39]. Rather than eliciting merely strong or weak responses to a single emotion, composite emotional stimulation enables the integration of dynamic neural fluctuations, which can be transformed into quantitative feature indicators, thereby improving the accuracy of depression recognition.

### 3.4. Ablation Experiment

**Figure 8:**
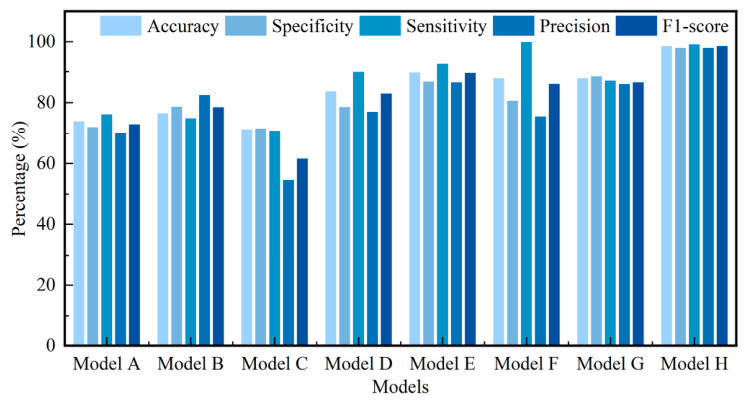
Analysis of ablation experiment results bar graphs.

To validate and demonstrate the necessity of the key technical components in this study, we designed multiple model variants and conducted systematic ablation experiments. Specifically, we compared the performance of the proposed model against counterparts with individual components removed. The results are presented in Figure 8, encompassing eight distinct configurations: Model A: Basic model, general GCN. Model B: TPGCN model. Model C: GAN model. Model D: Transformer model. Model E: TPGCN + GAN model. Model F: GAN + transformer model. Model G: TPGCN + transformer model. Model H: Our proposed model.

As illustrated in Figure 8, the detection accuracy of the model declines to varying degrees upon removal of different modules, confirming the essential contribution of each component within the proposed architecture. Notably, the absence of the TPGCN module leads to a more pronounced reduction in accuracy compared to the exclusion of the transformer module, underscoring the pivotal role of deep spatial features in EEG signal processing.

**Figure 9:**
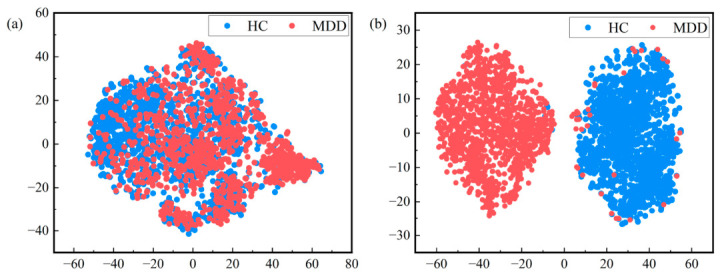
Characterization of model inputs and outputs using the t-SNE approach. (**a**) Model input features. (**b**) Model output features.

The input and output features of the model are visualized using t-stochastic Neighbor Embedding (t-SNE) to evaluate the feature extraction capability of the proposed model. The t-SNE maps high-dimensional data into a two-dimensional space by converting pairwise similarities between data points into probability distributions, as illustrated in Figure 9, which presents the distribution of input and output features. Compared with Figure 9a, the features in Figure 9b demonstrate markedly enhanced class separability, demonstrating that the DR-Net model effectively captures both intra-channel and inter-channel EEG information. Moreover, it shows strong capacity in integrating dynamic temporal and spatial features, thereby proving highly effective for the depression recognition and analysis.

### 3.5. Comparison with Existing Methods

To evaluate the performance of the DR-Net model and demonstrate the effectiveness of the proposed method, a comprehensive comparison was conducted against five baseline methods and eight existing state-of-the-art (SOTA) approaches. Table 3 presents the results in comparison with baseline classifiers, including Support Vector Machines (SVMs), Logistic Regression (LR), Naive Bayes, Decision Trees (DTs), and Two-Layer Neural Networks. The DR-Net model consistently outperforms all baseline methods across all classification metrics. Although SVM and DT achieve relatively high specificity, their sensitivity remains below 60%, indicating a tendency to misclassify positive cases and highlighting their limitations in achieving balanced and objective depression recognition.

Table 4 provides a comprehensive comparison between DR-Net and other related works in which deep learning techniques have been applied for depression classification based on EEG. A detailed evaluation is provided with respect to methodology and classification performance. The proposed model outperforms existing methods across all classification metrics, demonstrating balanced performance and consistent evaluation criteria for both depressed and healthy subjects.

### 3.6. Clinical Evaluation

**Figure 10:**
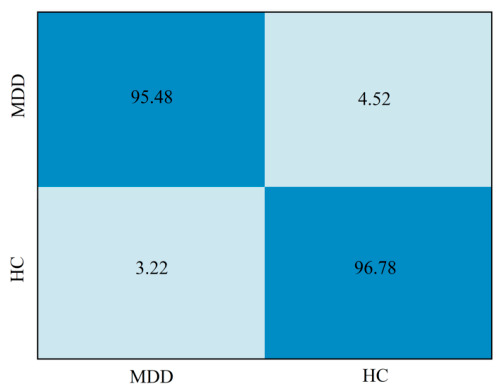
Percentage-based confusion matrix of DR-Net in clinical data.

To validate the applicability and effectiveness of the proposed model in clinical settings, this study applied the DR-Net model to EEG data collected from clinical subjects during emotional stimulation tasks for depression classification. The experimental results showed that the DR-Net achieved an accuracy of 96.13%, a specificity of 96.78%, a sensitivity of 95.48%, a precision of 96.74%, and an F1 score of 96.10% on clinical samples. The confusion matrix is presented in Figure 10. These results indicate that the spatio-temporal feature modeling framework integrating TPGCN and transformer can effectively capture the complex dynamic patterns of depression-related EEG signals and identify potential neurophysiological abnormalities. Moreover, data augmentation via GAN further enhances the generalization capability of the model under small-sample conditions, verifying the robustness and practical applicability of the DR-Net in real clinical settings. In summary, the DR-Net model proposed in this paper provides an efficient, scalable, and clinically viable solution for EEG-based MDD diagnosis.

## 4. Limitations and Discussions

Several limitations should be acknowledged in this study. First, potential demographic confounding factors may have influenced the EEG patterns and classification performance. Although participants were recruited according to predefined inclusion and exclusion criteria, demographic variables such as age, sex, and education level may affect EEG activity. Therefore, future studies with larger sample sizes and more demographically diverse cohorts are warranted to examine the potential influence of these factors on depression-related EEG alterations.

Second, although the proposed model was evaluated using the available dataset and an independent clinical cohort, further validation with larger external datasets is still needed. Data collected from different clinical centers, EEG acquisition systems, and populations would be valuable for assessing the generalizability, robustness, and reproducibility of the proposed method.

Third, the potential influence of medication status should be further considered. In this study, participants who had taken psychotropic medication within two weeks before the experiment were excluded. However, medication history and long-term medication effects may still influence EEG signals and depression-related neural activity. Future studies should collect more detailed medication information and examine medication-related effects.

Finally, this study focused primarily on prefrontal EEG features to reduce computational complexity and to emphasize brain regions closely associated with emotional regulation and depression. However, depression is a disorder involving distributed abnormalities across multiple interconnected brain regions [46]. Therefore, future work should consider incorporating EEG features from broader brain regions and investigating multi-region functional interactions to provide a more comprehensive characterization of depression-related neural mechanisms.

## 5. Conclusions

This paper proposes a novel depression identification model, DR-Net, based on dynamic spatiotemporal feature extraction. Comprehensive evaluations on the MODMA dataset and in the clinical validation yield promising results. The model effectively leverages the spatial topological structure of the brain network and the long-term temporal dependencies of EEG. And the approach employs a targeted input strategy centered on prefrontal cortex-associated neural channels, thereby reducing computational overhead without compromising task-relevant discriminative information. The proposed TPGCN enables the capture of dynamic topological dependencies among channels, combined with the Transformer to model long-range temporal relationships, and facilitates the interactive exchange of temporal and spatial information of EEG. Furthermore, the method integrates multi-channel spatiotemporal attributes with multi-task experimental paradigms, analyzes depression recognition performance from multiple emotional stimulation perspectives, and confirms its effectiveness and practical applicability through interpretability analysis, providing the possibility for the clinical auxiliary application of the model in real scenarios.

## Figures and Tables

**Table 1:** Description of Database Details.

Dataset	MODMA		Clinical Data	
Status	Patients	HC	Patients	HC
Cases (N)	24	29	26	26
Gender (male/female)	11/13	9/20	7/19	9/17
Age (years)	30.88 ± 10.37	31.45 ± 9.15	39.88 ± 15.94	38.88 ± 14.08
Education (years)	13.25 ± 3.78	15.96 ± 2.32	—	—
Score of PHQ-9	19.33 ± 3.50	2.66 ± 1.80	—	—
Score of HAMD	—	—	19.96 ± 5.25	4.81 ± 1.10
Number of channels	128	128	43	43
Sampling frequency	250 Hz	250 Hz	1000 Hz	1000 Hz

**Table 2:** Time and Frequency Domain Feature Calculation Formulas.

Domain	Features	Computational Formula
Time domain	Mean	$\mu =\frac{1}{N}\sum_{i=1}^{N}x_{i}$
	Standard deviation	$\sigma =\sqrt{\frac{1}{N}\sum_{i=1}^{N}{\left(x_{i}-\mu \right)}^{2}}$
	Variance	$\sigma^{2}=\frac{1}{N}\sum_{i=1}^{N}{\left(x_{i}-\mu \right)}^{2}$
	Skewness	$s=\frac{\frac{1}{N}\sum_{i=1}^{N}{\left(x_{i}-\mu \right)}^{3}}{{\left(\frac{1}{N}\sum_{i=1}^{N}{\left(x_{i}-\mu \right)}^{2}\right)}^{3/2}}$
	Kurtosis	$k=\frac{\frac{1}{N}\sum_{i=1}^{N}{\left(x_{i}-\mu \right)}^{4}}{{\left(\frac{1}{N}\sum_{i=1}^{N}{\left(x_{i}-\mu \right)}^{2}\right)}^{2}}-3$
	Hjorth	$activity=\sigma^{2}$ $mobility=\sqrt{\frac{{activity}_{x’}}{{activity}_{x}}}$ $complexity=\sqrt{\frac{{mobility}_{x’}}{{mobility}_{x}}}$
Frequency domain	Spectral analysis	$F\left(\omega \right)=\int_{-\infty }^{+\infty }x(t)e^{-j\omega t}dt$
	Power spectral density	$PSD\left(\omega \right)=\underset{T\to \infty }{\lim}\frac{{F(\omega )}^{2}}{T}$

**Table 3:** Comparison of the Basic Methods for Depression-EEG Classification.

Methods	Acc	Spe	Sen	Pre	F1
SVM	76.36	96.5	52.01	92.49	66.58
LR	69.34	78.14	58.71	68.98	63.43
Plain Bayes	59.51	64.48	53.52	55.49	54.49
Decision tree	73.47	86.27	58.0	77.76	66.44
BNN	82.54	84.79	79.81	81.29	80.54
DR-Net	98.7	98.15	99.26	98.16	98.71

**Table 4:** Comparison of the SOTA Methods for Depression-EEG Classification.

Methods	Acc	Spe	Sen	Pre	F1	Ref
MGSN	89.56	93.67	85.44	93.11	89.11	[25]
EDT	92.25	89.67	94.83	91.18	92.66	[40]
LSTM+DNN+MLR	89.70	87.50	90.80	93.50	92.10	[41]
SGP-SL	84.91	82.76	87.50	80.77	84.00	[42]
Multilayer network	86.88	81.16	95.12	77.80	85.59	[43]
SSPA-GCN	92.87	93.59	92.00	92.23	92.12	[9]
HEMAsNet	80.67	72.79	84.13	78.24	81.08	[44]
DepL-GCN	81.13	88.24	76.01	89.98	79.81	[45]
DR-Net	98.7	98.15	99.26	98.16	98.71	

## Data Availability

The data are not publicly available due to privacy or ethical restrictions. The datasets used and analyzed during the current study are available from the corresponding author upon reasonable request.
